# Supplementary material for: Compartmentalization-induced phosphorescent emission enhancement and triplet energy transfer in aqueous medium
Source: Nat Commun. 2019 Aug 19;10:3735. doi: 10.1038/s41467-019-11650-7 (PMC6700130; doi:10.1038/s41467-019-11650-7)
Supplement: Supplementary file 1 — Supplementary Information [file 41467_2019_11650_MOESM1_ESM.pdf]

Compartmentalization-induced phosphorescent emission enhancement and triplet energy transfer in aqueous medium

Z. Li *et al.*

Supplementary Methods

Reagents and reactants: Copper(I) iodide (CuI), 4-iodophenol, trimethylsilyl acetylene, 4-methylbenzenesulfonyl chloride, Brij-S20, and poly(ethylene glycol) monomethyl ether (CH_3O-PEG_{2000}-OH, M_w~2,000) were reagent grade and used as received. 4-Hydroxyphenylacetylene, Au(C^N^C)Cl and compounds **1**–**2** were synthesized according to the previously reported procedures.[1-3] Other reagents and solvents were employed as purchased.

Theoretical calculations: DFT computations were performed by utilizing Gaussian 09 D.01 software package.[4] During the optimization process, Lanl2dz core potential was chosen to describe Pt and Au atoms, while all other elements were described by using ωb97xd and 6-31G as dispersion corrected exchange functional and basis set, respectively.

*Synthesis of compound **4**:*

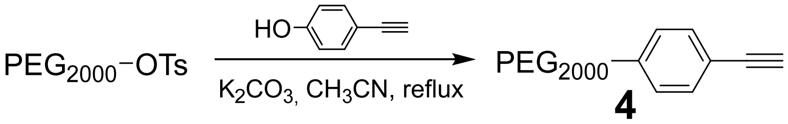

PEG_{2000}-OTs (2.00 g, 0.92 mmol), 4-hydroxyphenylacetylene (0.16 g, 1.35 mmol), K_2CO_3 (0.38 g, 2.76 mmol) were dissolved in 50 mL of CH_3CN and refluxed for 16 hours. After cooling to room temperature, the mixture was filtered through the celite pad. The filtrate was evaporated under reduced pressure, and the residue was purified by flash column chromatography (SiO_2, CH_3OH/CH_2Cl_2, 1 : 40 *v*/*v* as the eluent) to provide **4** as a white solid (1.68 g, 87%). ^{1}H NMR (400 MHz, $CDCl_3$, room temperature, Supplementary Figure 23) δ (ppm): 7.34 (d, J = 8.8 Hz, 2H), 6.79 (d, J = 8.8 Hz, 2H), 4.08–4.05 (m, 2H), 3.80–3.77 (m, 2H), 3.58 (s, 190H), 3.31 (s, 3H), 2.94 (s, 1H).

*Synthesis of compound **3***

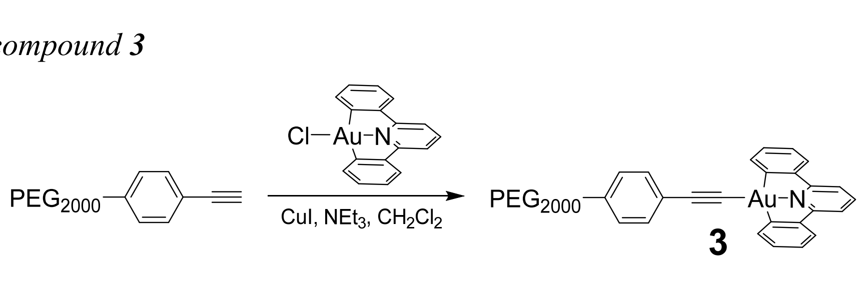

Compound **4** (0.80 g, 0.38 mmol), Au(C^N^C)Cl (0.23 g, 0.50 mmol), CuI (0.02 g, 0.01 mmol), and NEt_3 (4 mL) in CH_2Cl_2 (40 mL) were stirred at room temperature for 48 hours.

The mixture was evaporated under reduced pressure, and the residue was purified by column chromatography (SiO_2, CH_3OH/CH_2Cl_2, 1 : 40 *v*/*v* as the eluent) to afford **3** as a pale-yellow solid (0.88 g, 92%). ^1H NMR (300 MHz, $CDCl_3$, room temperature, Supplementary Figure 24) δ (ppm): 8.10 (d, J = 7.2 Hz, 2H), 7.87 (t, J = 8.0 Hz, 2H), 7.56 (m, 4H), 7.49 (d, J = 8.0 Hz, 2H), 7.40 (t, J = 7.3 Hz, 2H), 7.25 (d, J = 7.5 Hz, 2H), 6.89 (d, J = 8.6 Hz, 2H), 4.18–4.14 (m, 4H), 3.90–3.85 (m, 4H), 3.64 (s, 186H), 3.38 (s, 3H). ^{13}C NMR (75 MHz, $CDCl_3$, room temperature, Supplementary Figure 25) δ (ppm): 166, 164, 157, 148, 141, 136, 132, 131, 126, 125, 118, 116, 114, 100, 89, 71, 70, 69, 67, 58, 45. MALDI−TOF−MS: M_n = 2444.68 g mol^{-1}, M_w = 2457.15 g mol^{-1}, PDI = 1.01 (Supplementary Figure 26).

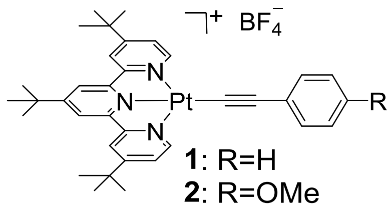

1: R=H
2: R=OMe

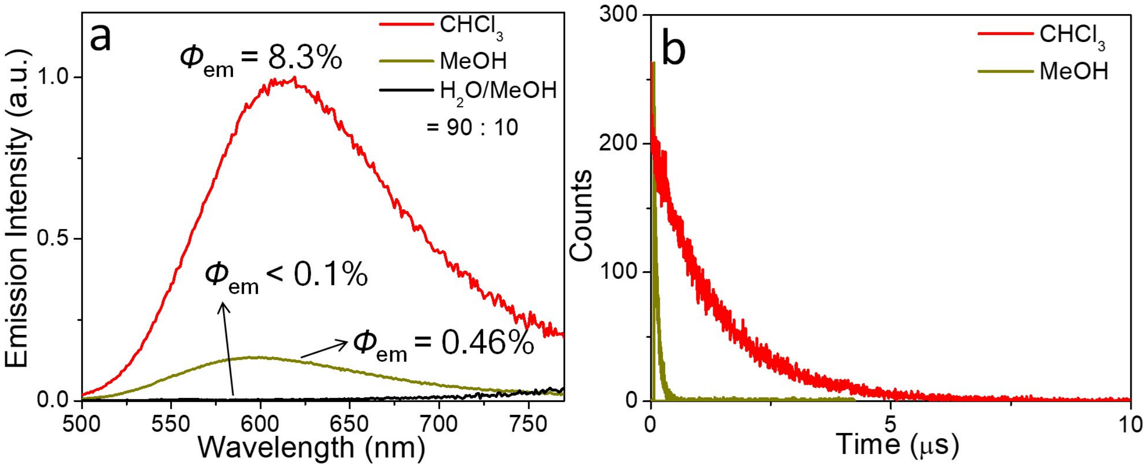

Supplementary Figure 1. Emission quenching behaviors of platinum(II) phosphor **1** in aqueous medium**.** a) Emission spectra (λ_{ex} = 470 nm) and b) emission decay traces of platinum(II) phosphor **1** (5.00×10^{-5} M). As can be seen, the emission intensity declines upon increasing the solvent polarity. When switching the solvent from chloroform to methanol, the lifetime decreases from 1.32 μs to 0.064 μs. No emission lifetime can be acquired for **1** in water/MeOH (90 : 10, *v*/*v*) , because of the negligible emission intensity [since **1** is not soluble in pure water, water/MeOH (90 : 10, *v*/*v*) is employed as an alternative].

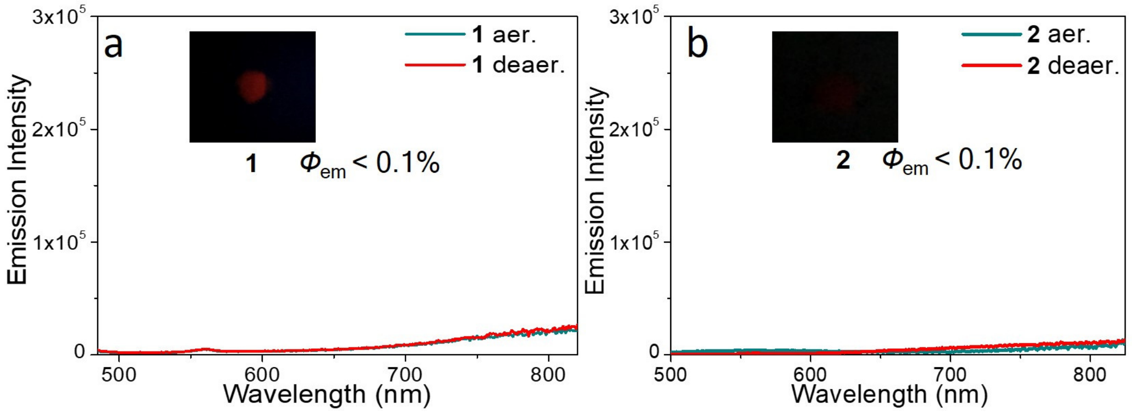

Supplementary Figure 2. Emission quenching behaviors of platinum(II) phosphors **1** and **2** in solid state**.** a) Emission spectra of the aerated (dark cyan line) and deaerated (red line) samples of **1** in water/MeOH (90 : 10, *v*/*v*). b) Emission spectra of the aerated (dark cyan line) and deaerated (red line) samples of **2** in water/MeOH (90 : 10, *v*/*v*). Inset of a–b): emission color images of **1** and **2** in the solid state under 365 nm UV lamp. No emission enhancement is observed for **1** and **2** in water/MeOH (90 : 10, *v*/*v*) under the deaerated condition (Φ_{em} < 0.1%). Hence, self-aggregation caused quenching is the main reason for the emission intensity decrease of platinum(II) complexes. The conclusion can be further supported by the quenching of emission for both **1** and **2** in the solid state.

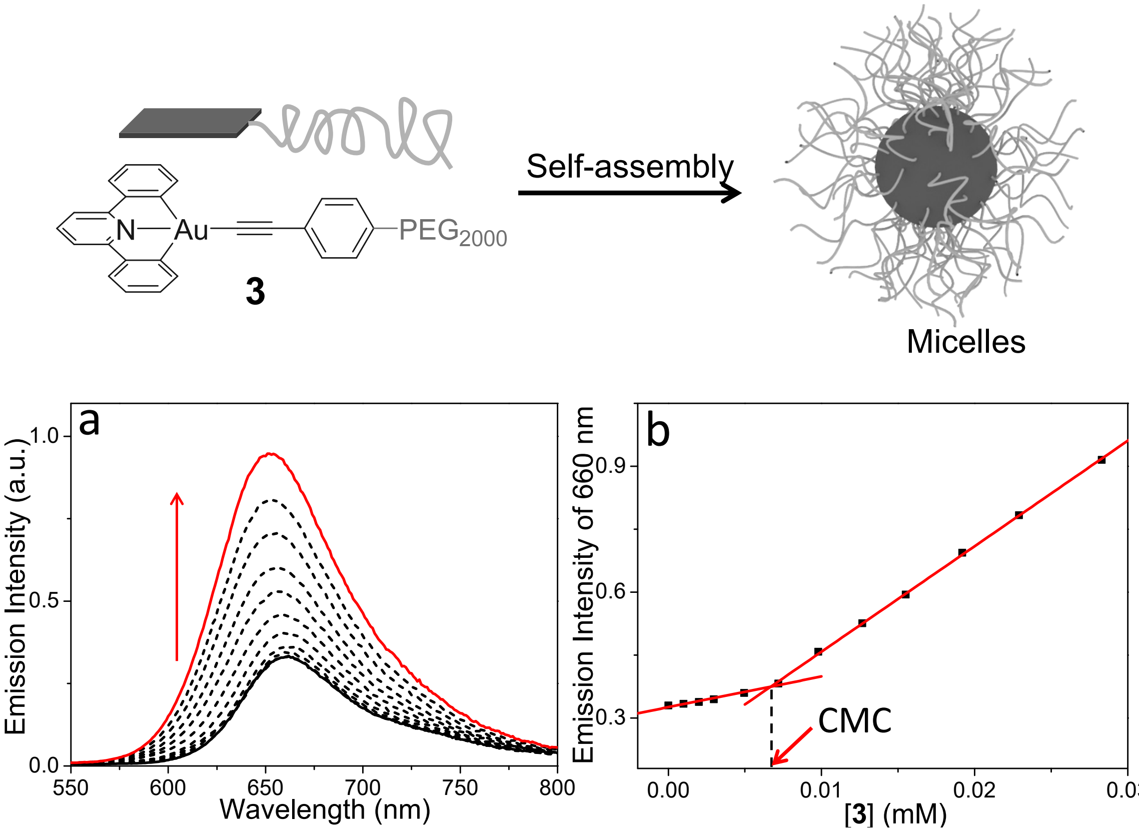

Supplementary Figure 3. Nile red encapsulation experiment for **3** in water. a) Fluorescent spectra (λ_{ex} = 525 nm) of Nile red (1 μM) in water. The arrow indicates the spectral change upon gradual addition of **3**. b) Relationship between the emission intensity at 660 nm and the concentration of **3**. The critical micelle concentration (CMC) of **3** is determined to be 6.7×10^{-6} M.

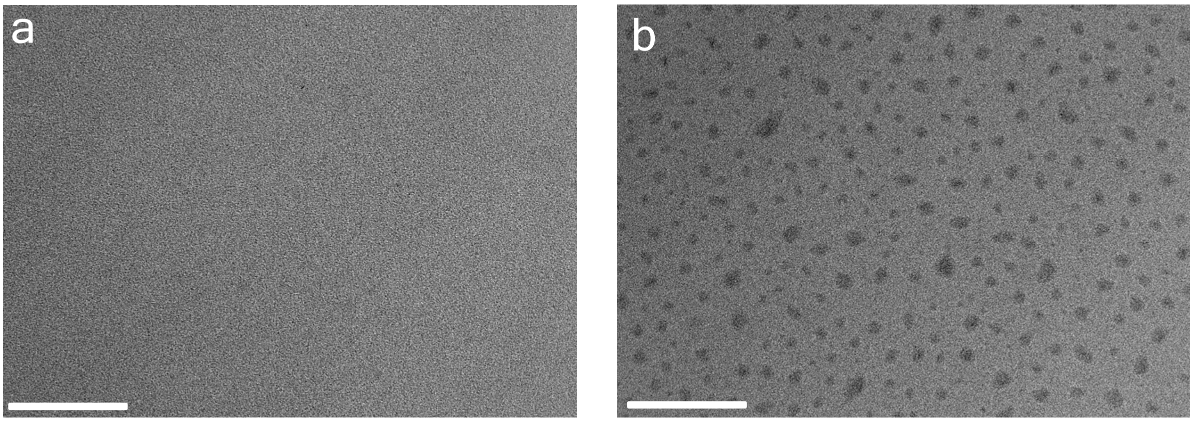

Supplementary Figure 4. Transmission electron microscopy (TEM) measurements for **3**. TEM images recorded on a copper grid by drop-casting a 5.00×10^{-5} M solution of **3**: a) in MeOH; and b) in water. As can be seen, **3** tends to form micelle structures in water, while no aggregates can be observed in MeOH under the same conditions. Scale bars: 500 nm.

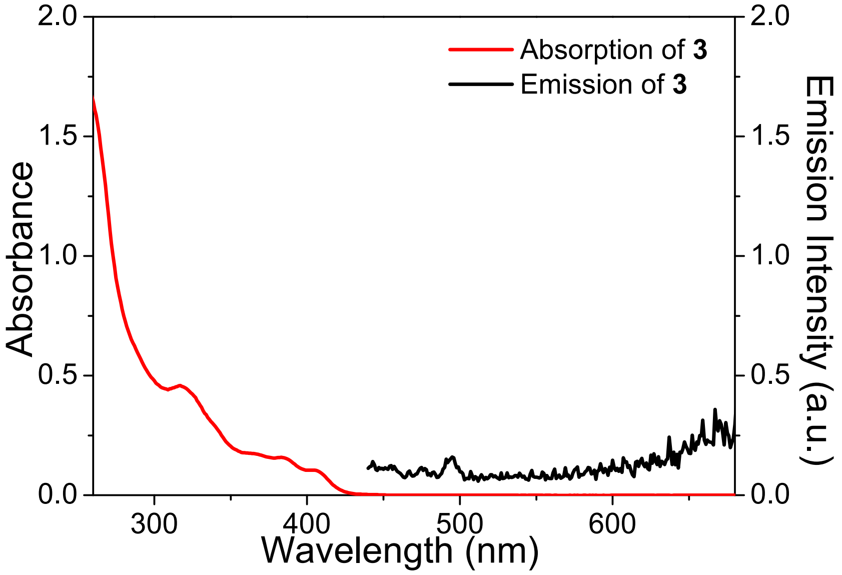

Supplementary Figure 5. Spectroscopic measurements of **3** in water. UV–Vis absorption (red line) and emission (black line) spectra of **3** (5.00×10^{-5} M in water). No obvious emission signal can be detected for **3**. It is mainly ascribed to the presence of thermally-accessible d-d or ligand-to-ligand charge transfer states for the [Au(III)(C^N^C)(C≡C–R)] unit on **3**.

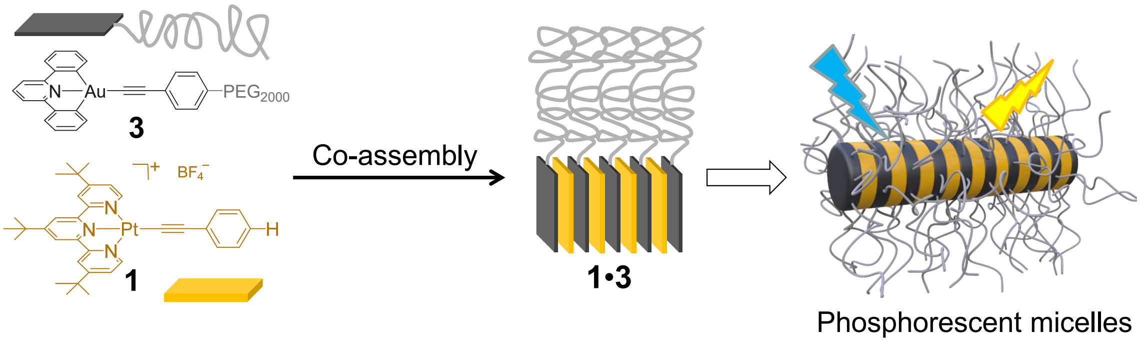

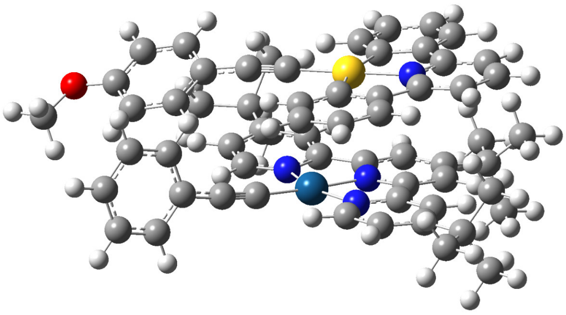

Supplementary Figure 6. Optimized structure of **1•3** *via* DFT calculation.[4] To reduce the computational costs, the PEG2000 side chain on **3** is replaced by the methoxyl group. As can be seen, inter-planar distance between [Pt(II)(N^N^N)] and [Au(III)(C^N^C)] units is determined to be 3.44 Å. It suggests the involvement of hetero π–π stacking interactions. Metal---metal interactions are absent, as evidenced by the long Pt(II)–Au(III) distance (3.69 Å) in the optimized geometry.

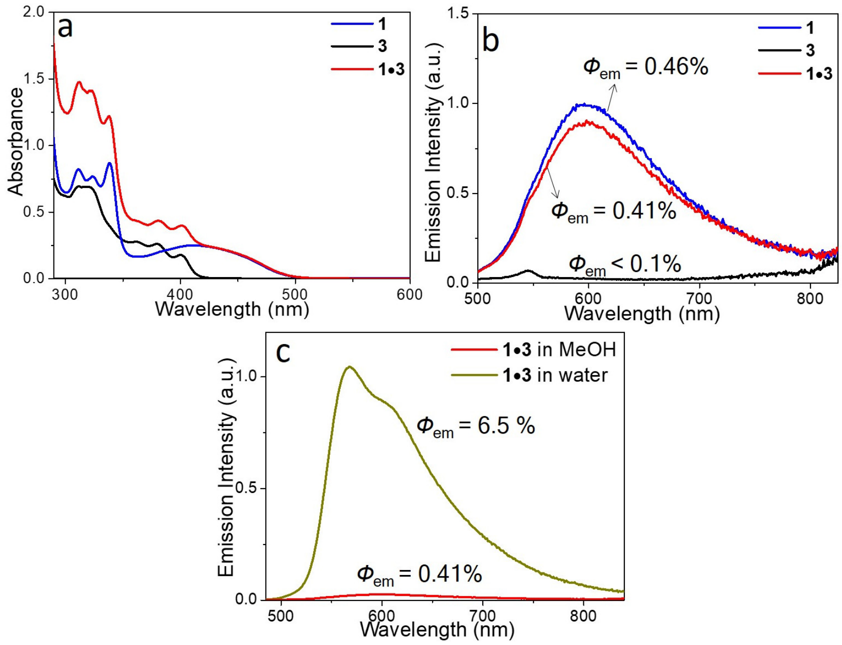

Supplementary Figure 7. Co-assembly behaviors between **1** and **3** in pure methanol. a) Absorption and b) emission spectra of **1** (blue line), **3** (black line) and **1•3** (red line) in pure methanol (5.00×10^{-5} M for each compound). The spectroscopic signals for **1•3** in pure methanol almost overlap with those of the individual species. Hence, it suggests the absence of non-covalent complexation between **1** and **3** in MeOH. c) Comparison of emission intensity of **1•3** in methanol and water (5.00×10^{-5} M for each compound). The emission quantum yield for **1•3** in water is 16-fold higher than that in methanol. The trend is opposite to that of the individual compound **1** (Φ_{em} = 0.46% in methanol, and less than 0.1% in water/MeOH (90 : 10, *v*/*v*), see Supplementary Figure 1). Hence, it validates that the **1•3** co-assembly is a prerequisite for the phosphorescent enhancement phenomenon.

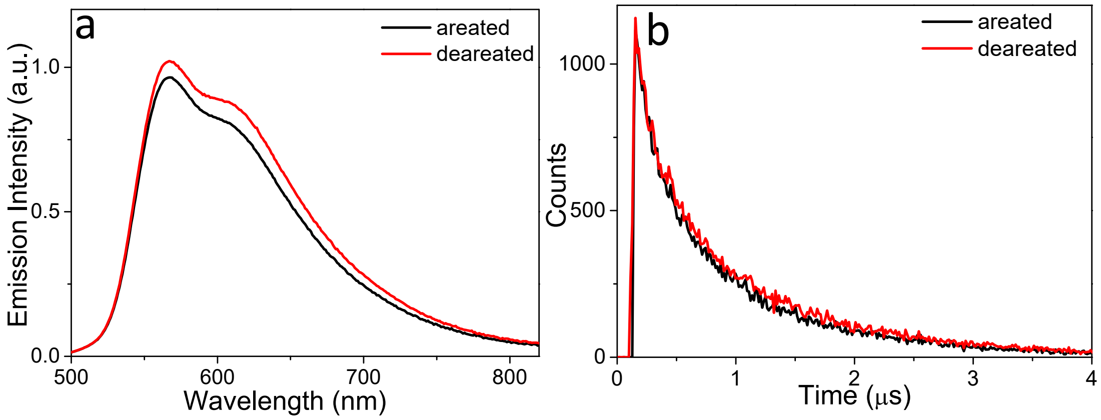

Supplementary Figure 8. Oxygen-sensitivity for the emission of complex **1•3** in aqueous medium. a) Emission spectra (λ_{ex} = 470 nm) and b) emission decay traces of complex **1•3** [5.00 × 10^{-5} M, water/methanol (90 : 10, *v*/*v*)] in the aerated (black line) and deaerated (red line) conditions. As can be seen, both the emission intensity and lifetime hardly change between the aerated (Φ_{em} = 0.061, τ = 0.67 μs) and deaerated (Φ_{em} = 0.065, τ = 0.69 μs) samples. Such phenomena suggest the insensitivity of **1•3** emission signal to the dissolved molecular oxygen.

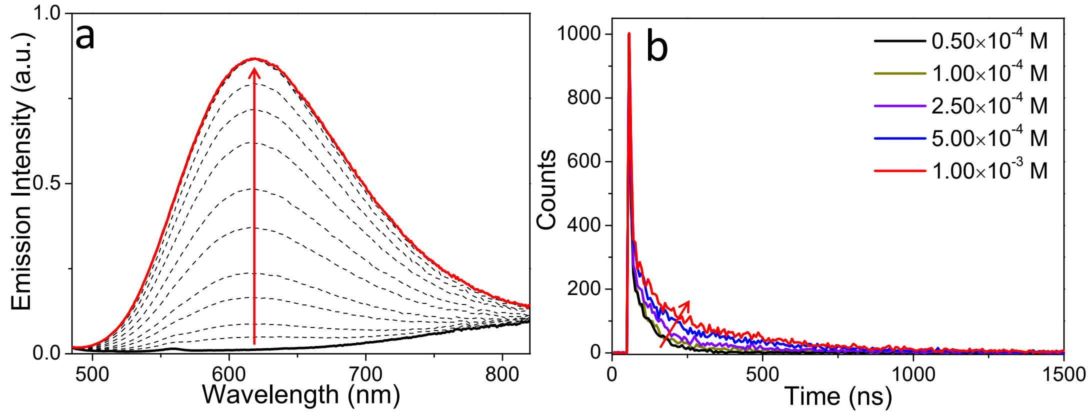

Supplementary Figure 9. Emission enhancement behaviors of Brij S20•**1** in aqueous medium. a) Emission spectra, and b) emission decay traces of **1** (5.00 × 10^{-5} M) in water/methanol (90 : 10, *v*/*v*), with the gradual addition of Brij S20 (concentration: ranging from 5.00 × 10^{-5} M to 1.00 × 10^{-3} M). When 20 equivalents of Brij S20 are added, the emission intensity of **1** reaches to the maximum value (Φ_{em} = 0.012, τ = 0.13 μs). It suggests the encapsulation of **1** into the hydrophobic environment provided by the amphiphile Brij S20.

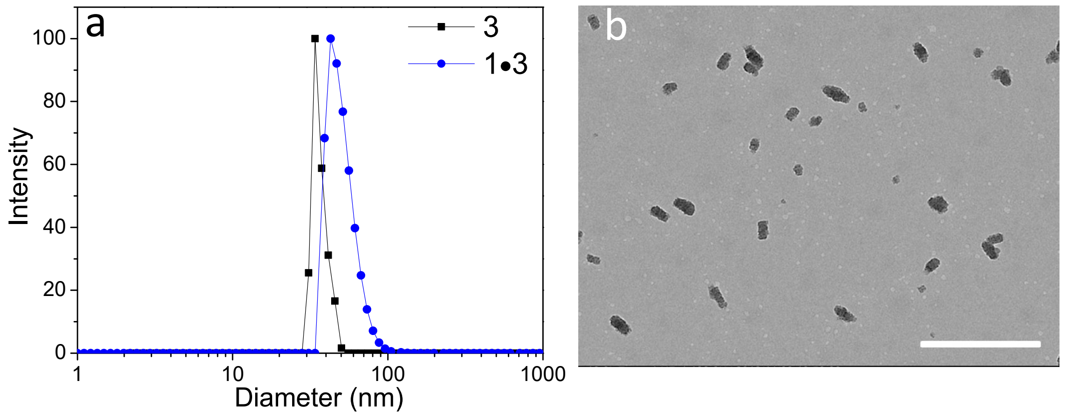

Supplementary Figure 10. Morphological characterization of complex **1•3** in water. a) Number-averaged DLS and b) TEM image for complex **1•3** [5.00 × 10^{-5} M in water]. Scale bar: 500 nm. According to the DLS measurement, the hydrodynamic diameter exhibits a slight increase from 34 nm for **3** (black line) to 41 nm for complex **1•3** (blue line). TEM image shows the formation of rod-like micelles for complex **1•3**.

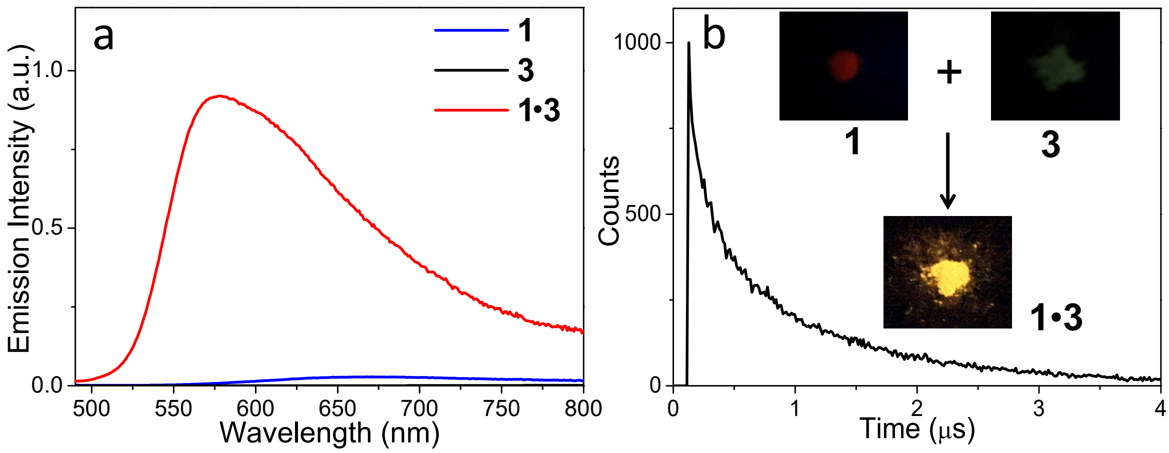

Supplementary Figure 11. Emission enhancement behaviors for complex **1•3** in the solid state. a) Emission spectra of **1**, **3**, and complex **1•3** in the solid state. b) Emission decay trace of complex **1•3** in solid state. Inset: emission color images of **1**, **3** and complex **1•3** in the solid state under 365 nm UV lamp. As can be seen, both **1** and **3** display very weak emission intensity in the solid state. Interestingly, upon mixing the equimolar amount of **1** and **3** and grinding them for 5 min, an intense yellow emission signal appears (the peak maximum is centered at 577 nm, while the lifetime is 0.62 μs). The emission enhancement phenomenon of complex **1•3** in the solid state is consistent with that in the aqueous medium [λ_{max} = 568 nm, τ = 0.69 μs in water/MeOH (90 : 10, *v*/*v*)]. Hence, it is evident that phosphorescent enhancement for complex **1•3** not only exists in the aqueous state, but maintains in the solid state.

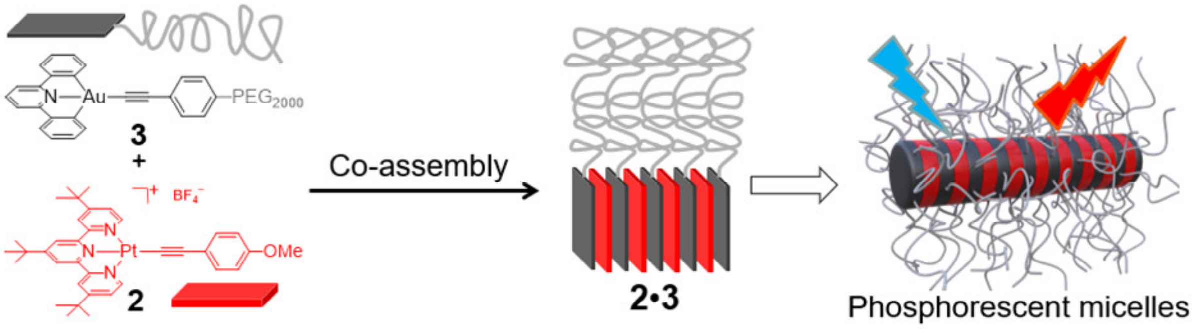

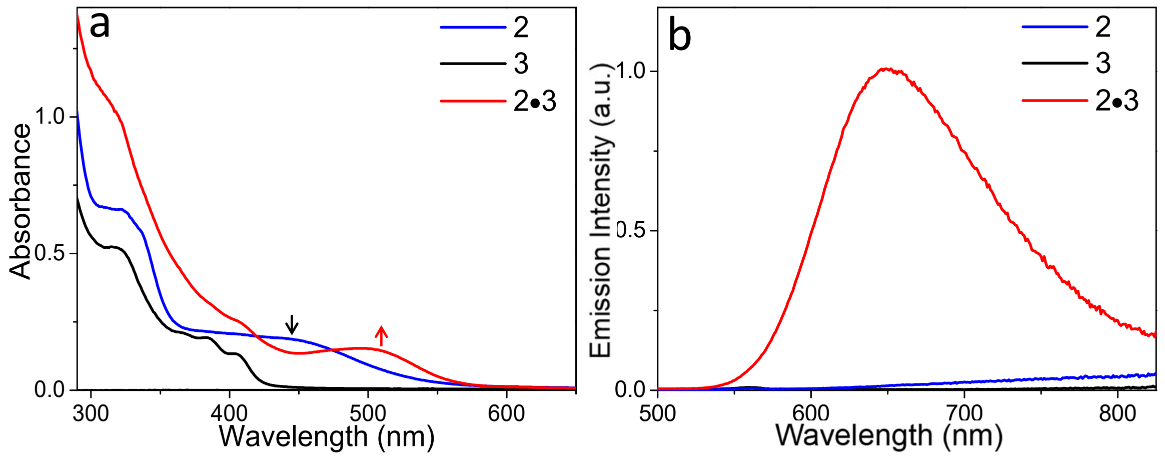

Supplementary Figure 12. Spectroscopic measurements of complex **2•3** in aqueous medium. a) UV/Vis absorbance and b) emission spectra of **2**, **3**, and complex **2•3** [5.00×10^{-5} M for each compound in water/methanol (90 : 10, *v*/*v*)]. The red-shifted MLCT/LLCT absorbance, together with the enhanced emission intensity, support the co-assembly between **2** and **3** in aqueous medium.

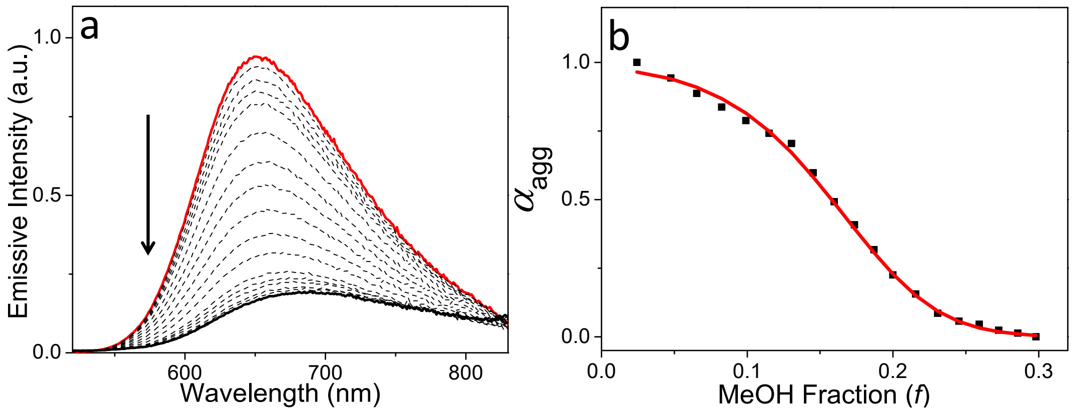

Supplementary Figure 13. Solvent-dependent emission spectra of **2•3**. a) Solvent-dependent emission spectra of **2•3** [5.00×10^{-5} M in water/MeOH (90 : 10, *v*/*v*)]. The arrow indicates the spectral changes upon increasing the MeOH fraction. b) α_{agg} as a function of MeOH volume fraction monitored at 648 nm. The red line denotes the mathematical fitting of the curve, according to the solvent-dependent equilibrium model. In detail, ΔG_0 (Gibbs free energy gain upon monomer association in pure water), *m* and cooperativity parameter (σ) values are determined to be –45.1 kJ mol^{-1}, 81.0 kJ mol^{-1} and 1, respectively. In water/MeOH (90 : 10, *v*/*v*), ΔG value for complex **2•3** is determined to be –37.0 kJ·mol^{-1}.

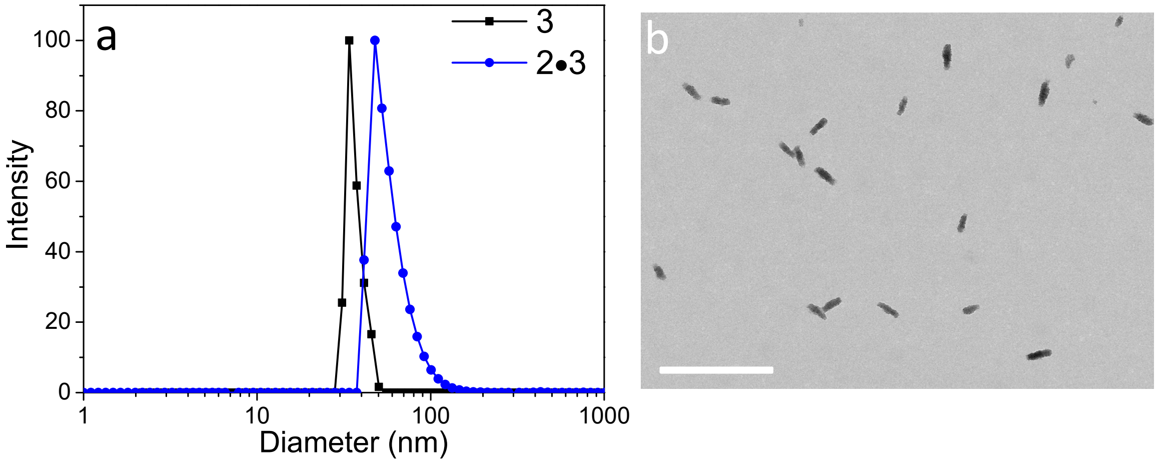

Supplementary Figure 14. Morphology characterization of complex **2•3** in water. a) Number-averaged DLS, and b) TEM image for complex **2•3** [5.00 × 10^{-5} M in water]. Scale bar: 500 nm. According to DLS measurement, the hydrodynamic diameter exhibits a slight increase from 34 nm for **3** (black line) to 45 nm for **2•3** (blue line). TEM image shows the formation of rod-like micelles for complex **2•3**.

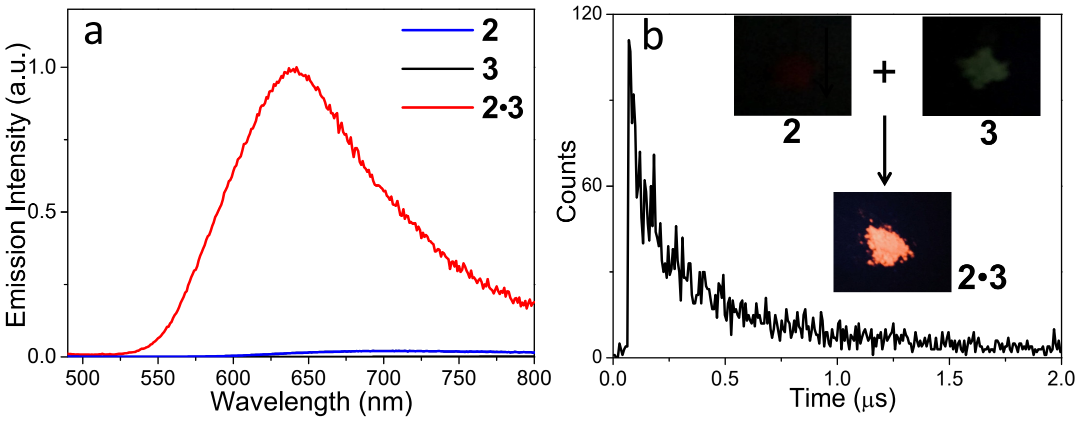

Supplementary Figure 15. Emission enhancement behaviors for complex **2•3** in solid state. a) Emission spectra of **2**, **3**, and complex **2•3** in solid state. b) Emission decay trace of complex **2•3** in solid state. Inset: emission color images of **2**, **3** and complex **2•3** in the solid state under 365 nm UV lamp. As can be seen, both **2** and **3** display very weak emission intensity in the solid state. Upon mixing the equimolar amount of **2** and **3** and grinding them for 5 min, an intense red emission signal appears (the peak maximum is centered at 641 nm, while the lifetime is 0.42 μs). The emission enhancement phenomenon of complex **2•3** in the solid state is consistent with that in the aqueous medium [λ_{max} = 648 nm, τ = 0.40 μs in water/MeOH (90 : 10, *v*/*v*)]. Hence, it is evident that phosphorescent enhancement for complex **2•3** not only exists in the aqueous state, but maintains in the solid state.

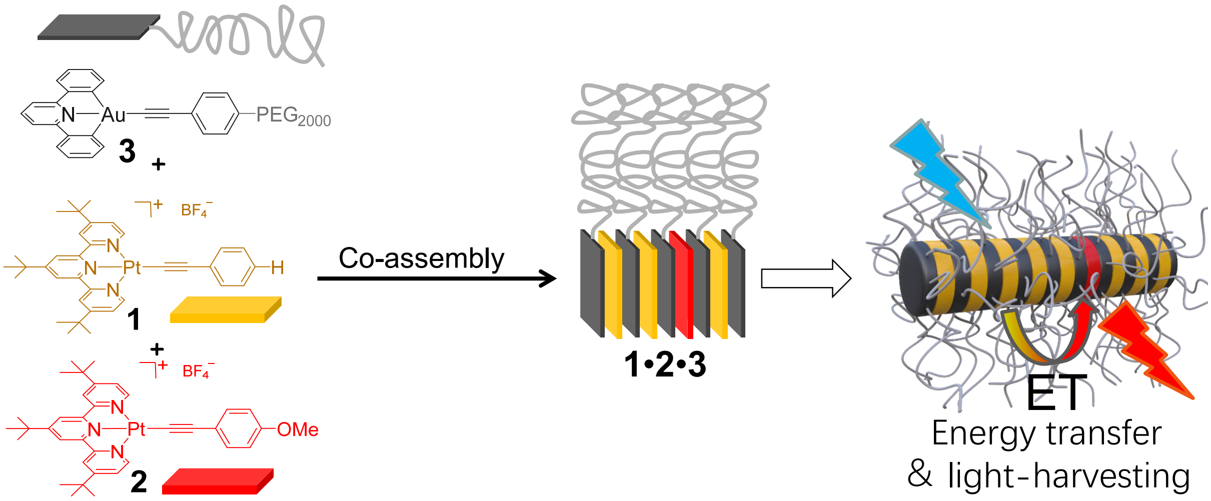

Calculation of the overlap integral(J): J is calculated according to Supplementary Equation (1).

$$J(\lambda)=\int_0^\infty \lambda^4 \times f_D(\lambda)\times \varepsilon_A(\lambda)\,d\lambda \qquad \textbf{Supplementary Equation (1)}$$

In the equation, λ is the wavelength (in cm), and $\varepsilon_A(\lambda)$ is the molar extinction coefficient of complex **2•3** (served as the energy transfer acceptor) at the specific wavelength λ. $f_D(\lambda)$ is the fraction of emission intensity of complex **1•3** (served as the energy transfer donor).

Overlap between the emission spectrum of donor and the absorption spectrum of acceptor is shown in Supplementary Figure 16. Accordingly, *J* value is calculated to be 2.07×10^{12} M^{-1} cm^{-1} nm^4, demonstrating the feasibility for energy transfer from **1•3** to **2•3**.

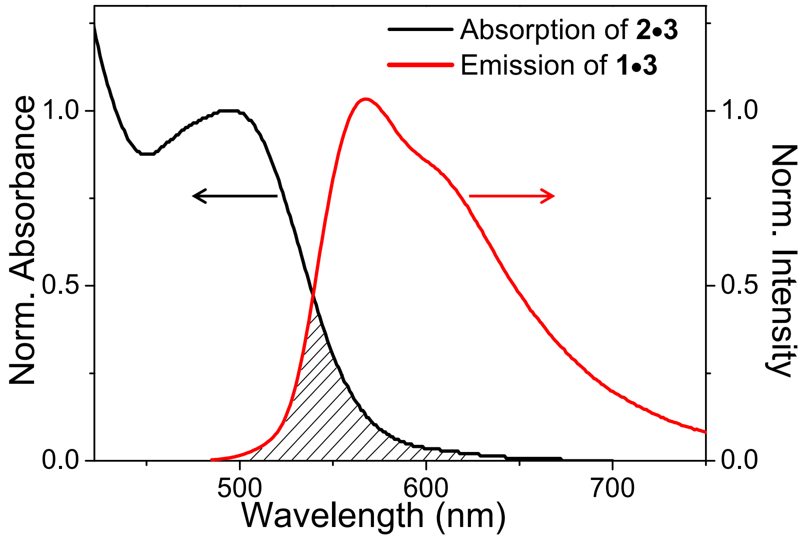

Supplementary Figure 16. Spectral overlap between **1•3** and **2•3**. Normalized emission spectra of **1•3** (red line), together with the normalized absorption spectra of **2•3** (black line).

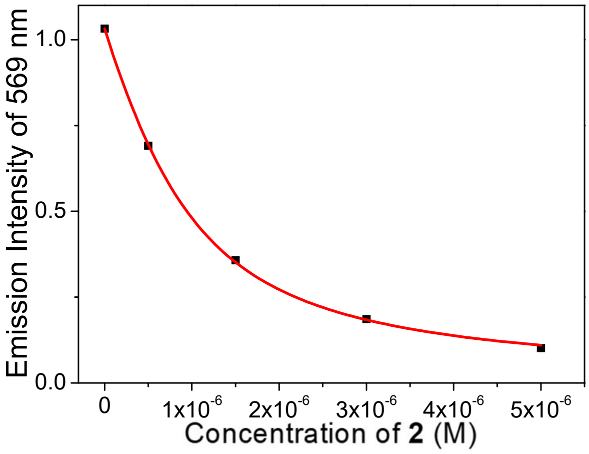

Supplementary Figure 17. Calculation of the donor number quenched by acceptor. Nonlinear least-squares fittings of the emission intensity of donor (I_D) versus the concentration of acceptor (C_A). The binding constant (K_a) and the concentration of $(D)_n$ are determined to be $(1.95 \pm 0.09) \times 10^6$ M^{-1} and $(7.67 \pm 0.59) \times 10^{-7}$ M, respectively. Since the concentration of donor (C_D) in the current system is 5.00×10^{-5} M, the n value is then calculated to be (65 ± 4).

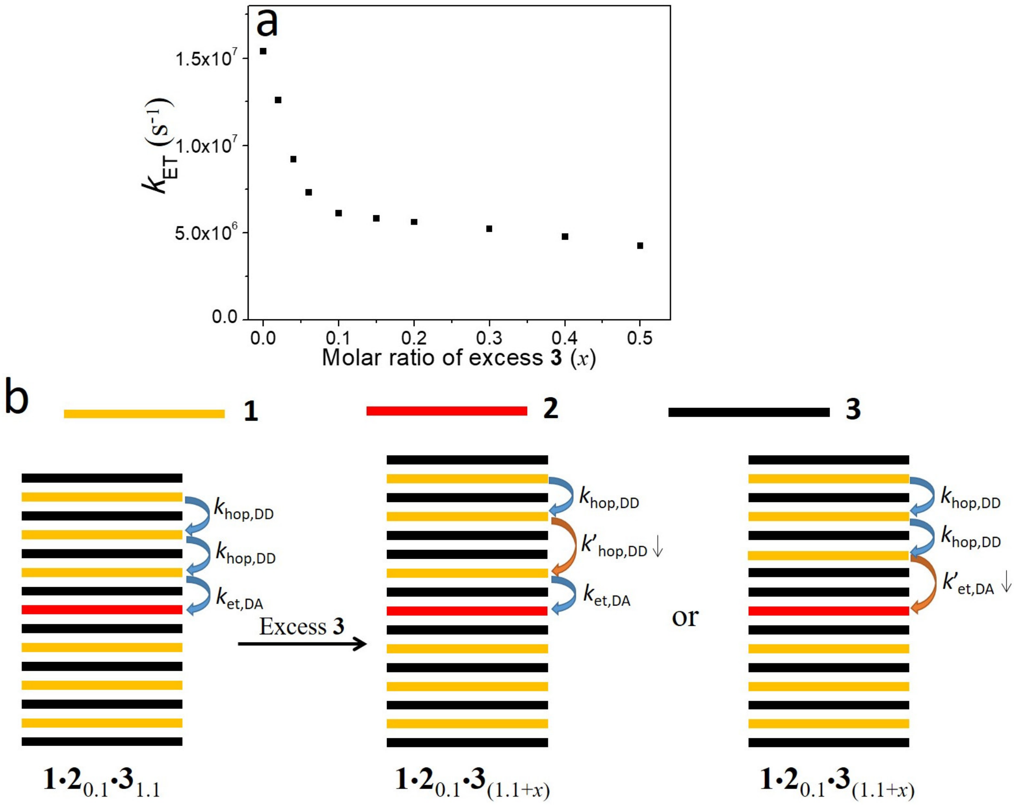

Supplementary Figure 18. Variation of energy transfer efficiency with different amount of **3**. a) Relationship of k_{ET} value in the ternary complex **1**•**2**$_{0.1}$•**3**$_{(1.1+x)}$ and the excessive amount of **3** [**1**: 5.00×10^{-5} M in water/MeOH (90 : 10, v/v), x varies from 0 to 0.5]. b) Schematic representation for the k_{ET} decrease mechanism. Addition of excessive amount of **3** ($x = 0.1$) into complex **1**•**2**$_{0.1}$•**3**$_{1.1}$ leads to 3-fold decrease of k_{ET} value. It is primarily ascribed to the random insertion of **3**, which increases the distance between the platinum phosphors. Apparently, compartmentalization exerts the crucial effect on the triplet energy transfer efficiency.

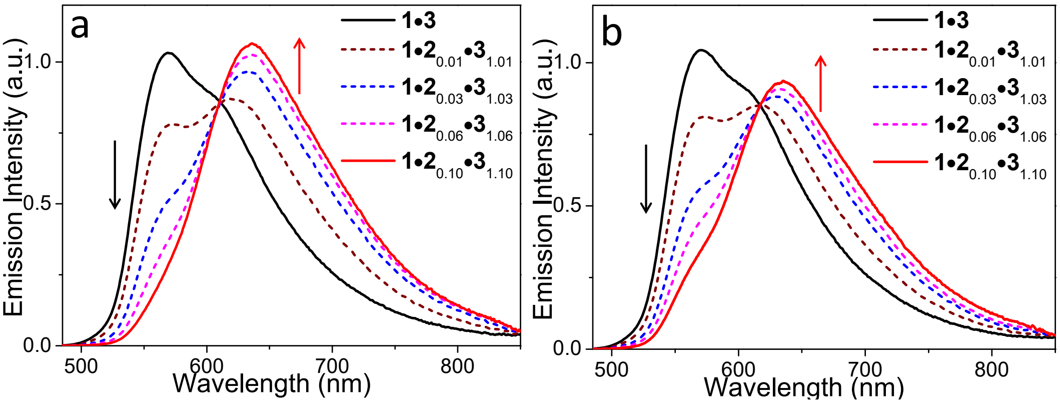

Supplementary Figure 19. Energy transfer dynamics of the ternary complex **1•2•3**. Emission spectra for the ternary complex **1•2**$_x$**•3**$_{(1+x)}$ [**1**: 5.00 × 10^{-5} M in water/MeOH (90 : 10, *v*/*v*), *x* varies from 0 to 0.1], by utilizing the two different prepared methods (see Figure 6a in the main text): a) method A; and b) method B. The Φ_{ET} values for method B are relatively lower than the corresponding ones obtained in method A, because of the different exchange dynamics in the ternary complex.

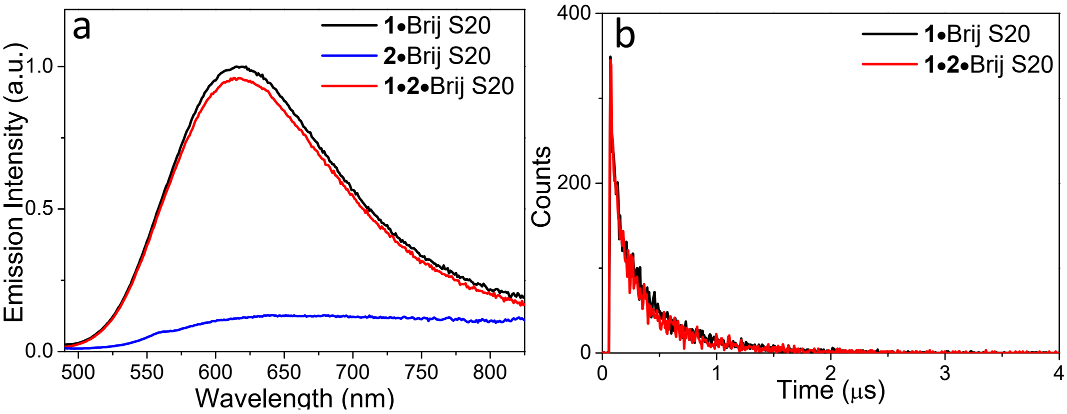

Supplementary Figure 20. Energy transfer of complex **1•2•**Brij S20 in aqueous medium. a) Emission spectra and b) emission decay traces of complex **1•2•**Brij S20 (5.00 × 10^{-5} for **1**, 5.00 × 10^{-6} for **2**, and 1.00 × 10^{-3} M for Brij S20) in water/methanol (90 : 10, *v*/*v*). No obvious ET takes place between **1** and **2** when replacing **3** with the surfactant Brij S20, highlighting the prominent role of the compartmentalized agent **3** for the high triplet ET efficiency.

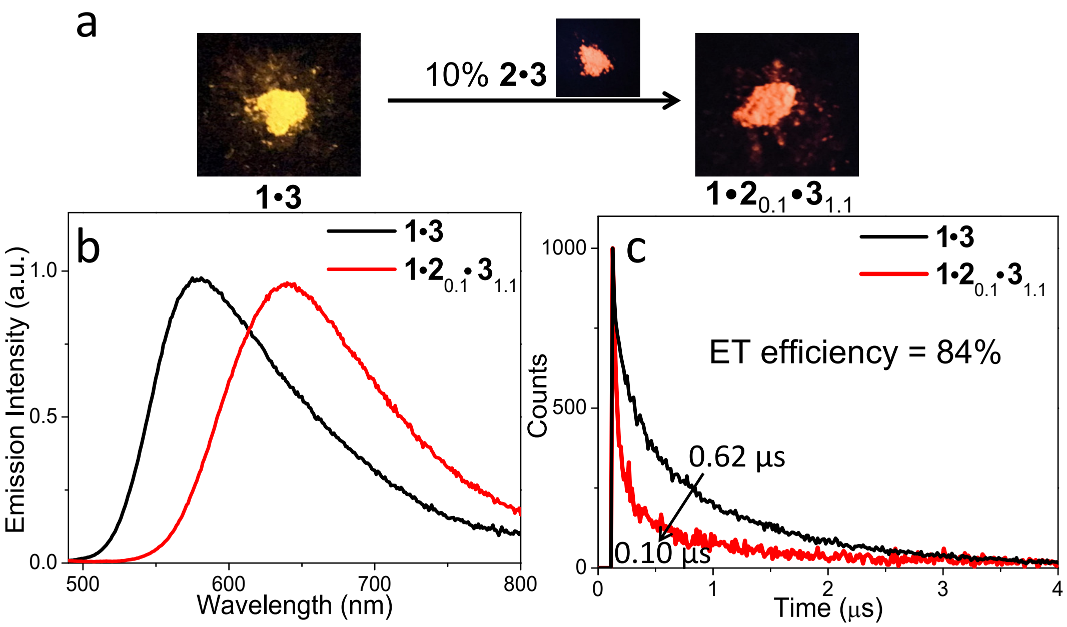

Supplementary Figure 21. Energy transfer of the ternary complex **1•2•3** in solid state. a) Emission color images of **1•2**$_{0.1}$**•3**$_{1.1}$ in the solid state under 365 nm UV lamp. To guarantee ideal donor–acceptor spatial organization in the ternary co-assembled system, one drop of methanol is added to the mixture of **1•3** and 10% amount of **2•3**. After grinding for 5 minutes, the mixed sample is further dried in vacuum to remove the solvent. b) Emission spectra, and c) emission decay traces of complex **1•3** and **1•2**$_{0.1}$**•3**$_{1.1}$ in the solid state. For lifetime measurements, the emission wavelength is chosen at 525 nm, considering that **2•3** displays negligible emission at the selected wavelength. As can be seen, the phosphorescent color changes from yellow of **1•3** to red of **1•2**$_{0.1}$**•3**$_{1.1}$. Moreover, the decay lifetime shortens from 0.62 μs of **1•3** to 0.10 μs of **1•2**$_{0.1}$**•3**$_{1.1}$. The energy transfer efficiency is determined to be 84% for **1•2**$_{0.1}$**•3**$_{1.1}$ in the solid state, which is comparable to the same sample in aqueous environment [91% in water/MeOH (90 : 10, *v*/*v*)]. Overall, it unambiguously supports that triplet energy transfer maintains for the ternary complex **1•2•3** in the solid state.

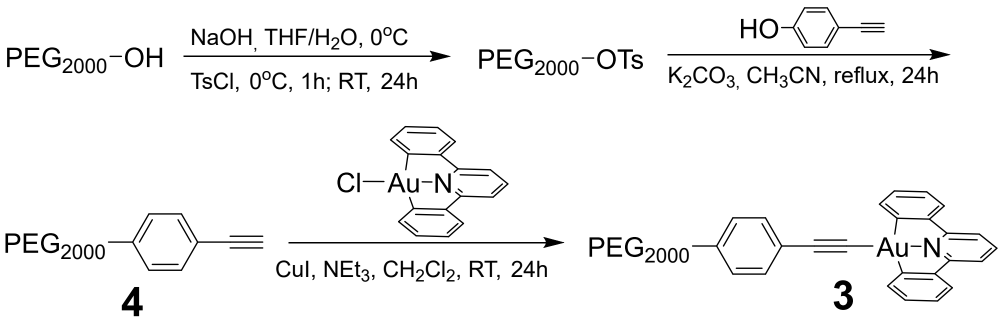

Supplementary Figure 22. Synthetic route to the rod–coil amphiphile **3**.

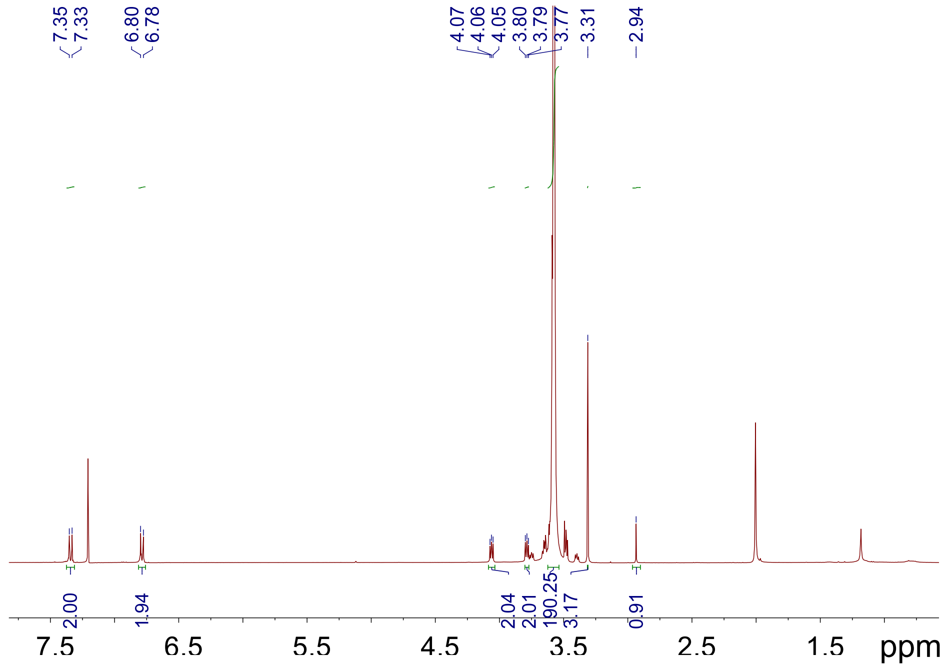

Supplementary Figure 23. ^{1}H NMR spectrum (400 MHz, $CDCl_3$, 298K) of **4**.

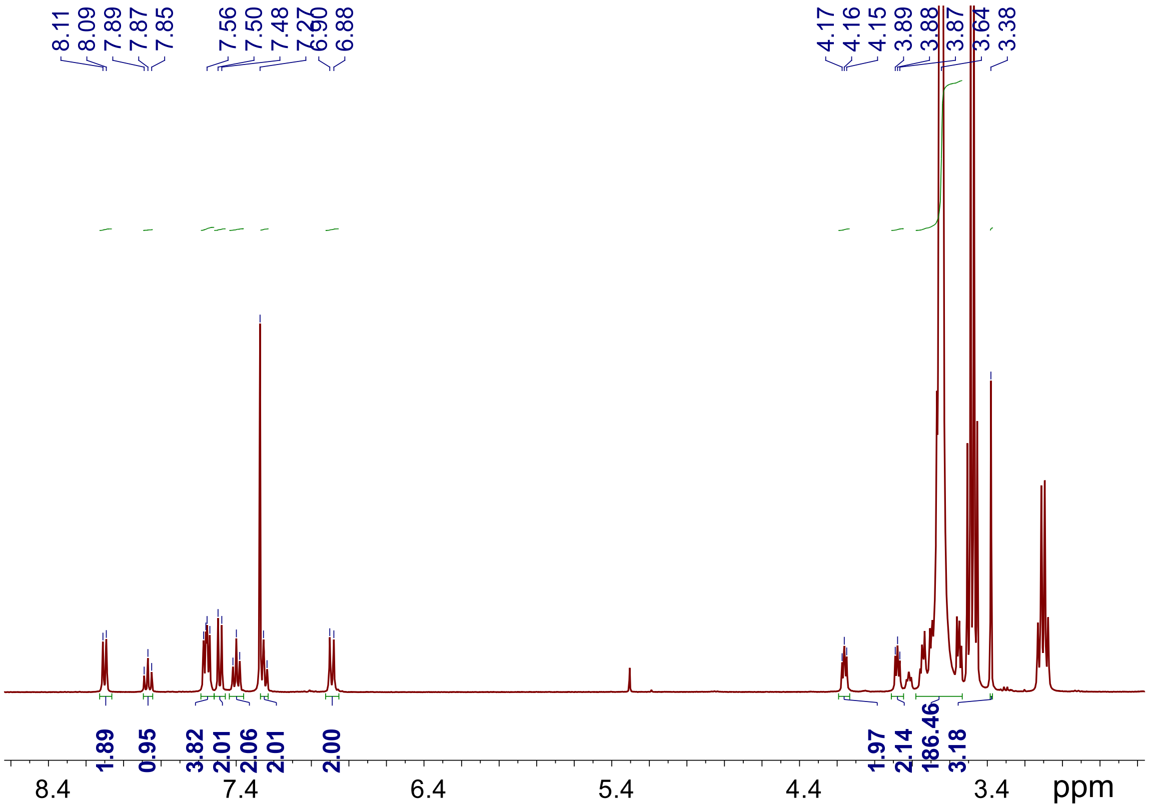

Supplementary Figure 24. ^{1}H NMR spectrum (400 MHz, $CDCl_3$, 298 K) of **3**.

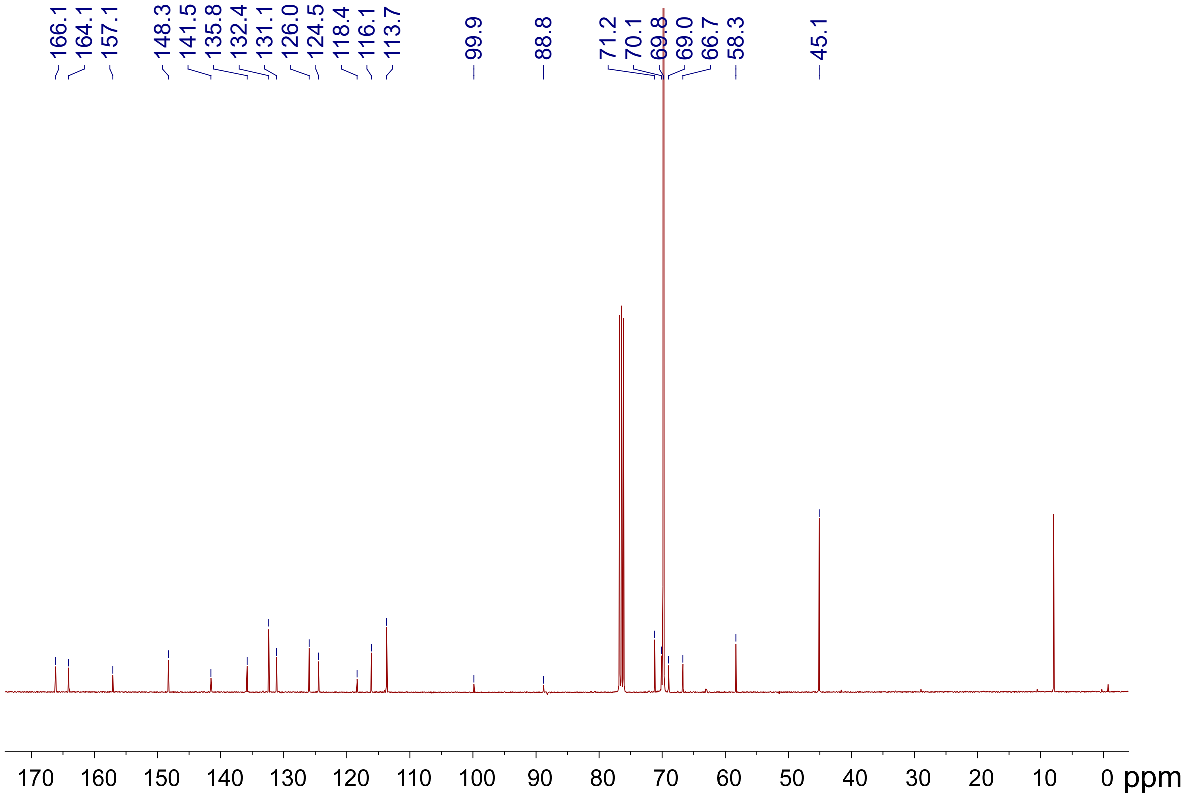

Supplementary Figure 25. ^{13}C NMR spectrum (100 MHz, $CDCl_3$, 298 K) of **3.**

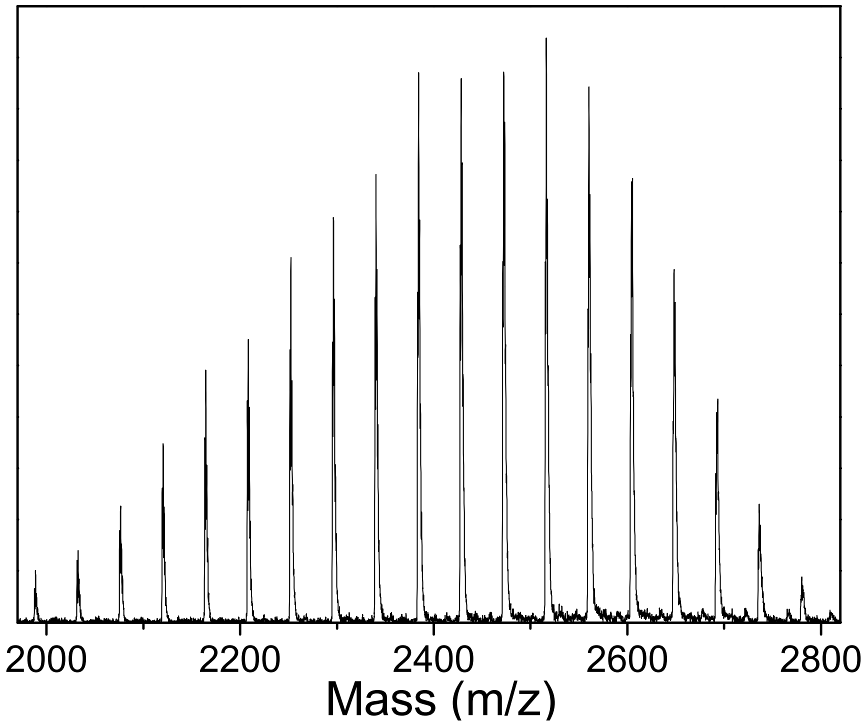

Supplementary Figure 26. MALDI−TOF−MS spectrum of **3**.

Supplementary References:

1. Lv, X. *et al.* Pre-organized molecular tweezer stabilized by intramolecular hydrogen bonds: solvent-responsive host–guest complexation. *Tetrahedron Lett.* **57**, 1971–1975 (2016).
2. To, W.-P. *et al.* Highly luminescent pincer gold(III) aryl emitters: thermally activated delayed fluorescence and solution-processed OLEDs. *Angew. Chem. Int. Ed.* **56**, 14036–14041 (2017).
3. Wang, X., Goeb, S., Ji, Z., Pogulaichenko, N. A. & Castellano, F. N. Homogeneous photocatalytic hydrogen production using π-conjugated platinum(II) arylacetylide sensitizers. *Inorg. Chem.* **50**, 705–707 (2011).
4. Frisch, M. J. *et al. Gaussian 09, Revision D.01* (*Gaussian, Inc., Wallingford CT, 2013*).
